# Thyroid Disorders in the Oncology Patient

**DOI:** 10.6004/jadpro.2015.6.2.2

**Published:** 2015-03-01

**Authors:** Kari Hartmann

**Affiliations:** University of Texas MD Anderson Cancer Center, Houston, Texas

## Abstract

Thyroid disease and cancer diagnoses are common conditions likely to coexist. Optimal management requires appropriate diagnostic testing and consideration of a number of factors, including overall health status and prognosis. Hypothyroidism and hyperthyroidism can lead to a number of symptoms that may affect not only quality of life but can interfere with the patient’s ability to tolerate cancer treatment. Imaging studies performed for cancer staging can identify incidental structural abnormalities in the thyroid, which should be assessed with dedicated neck ultrasonography and possibly fine-needle aspiration. Incidental thyroid cancer is most often less urgent than the patient’s presenting malignancy and can be addressed surgically when appropriate in the context of other treatments (i.e., chemotherapy). Providers working in an oncology setting, as well as primary care providers, should be aware of medications that are associated with hormonal abnormalities. Any patient with a history of neck or brain radiation therapy is at risk of developing hypothyroidism and possibly other endocrinopathies. Complex or very ill patients may benefit from a multidisciplinary approach that utilizes the experience of a knowledgeable endocrinologist.

Thyroid hormones regulate metabolic processes in the body, including the basal metabolic rate, nerve reflexes and conduction time, temperature regulation, cardiac contractility and heart rate, and intestinal transit time, among others. The thyroid affects the physiologic function of nearly all organs and systems.

Production of the thyroid hormone is regulated by feedback of circulating blood levels of several hormones. The hypothalamus secretes thyrotropin-releasing hormone (TRH), which stimulates release of thyroid-stimulating hormone (TSH) from the anterior pituitary. The TSH stimulates synthesis and release of thyroxine (T4) and triiodothyronine (T3) from the thyroid gland. Deiodination of T4 to T3 at the target organ also generates this biologically active hormone. Feedback of T3 to the hypothalamus and pituitary regulates secretion of TRH and TSH to maintain thyroid hormone levels within a narrow range (Shaw, Riedel, Burton, Field, & Feeley, 2005).

Nodules or masses may be present within the gland; some of them affect thyroid function, whereas many do not. Many structural thyroid abnormalities are detected incidentally on imaging studies. All of these issues must be addressed appropriately in the context of treatment for malignancy.

## SCOPE OF THE PROBLEM

Thyroid disease affects up to 1 in 20 people in the United States, and there is a 3:1 incidence ratio of females to males (Toft & McIver, 2007). Maintaining euthyroid status requires proper functioning of all regulatory steps. Several conditions can disrupt one or more of these steps, resulting in a thyroid disorder. These conditions are quite common, with 12% of elderly patients affected by hypothyroidism (Shaw et al., 2005). Symptoms may be vague or absent or may be confused with side effects of therapy given to treat the patient’s underlying malignancy. The 1:2 lifetime odds of developing cancer in men and the 1:3 risk of developing cancer in women (American Cancer Society, 2014) should be considered, and clearly many individuals will have both a thyroid disorder and a current or previously treated malignancy.

Iodine is taken up in thyroid tissue as a precursor to thyroid hormone. One of the most common diagnostic studies ordered for cancer patients is a CT (computed tomography) scan, often done with intravenous iodinated contrast. This can complicate both imaging studies and management of thyroid disorders. Occasionally, administration of iodine contrast can cause an acute exacerbation of underlying Graves’ disease.

Many patients present with new or chronic thyroid issues in the context of a cancer diagnosis. Some of them are identified incidentally as a result of their cancer studies, whereas others may be a direct result of the therapy they receive. Proper diagnosis and management of thyroid disease are essential for optimizing outcomes during and after cancer treatment as well as optimizing quality of life.

Thyroid disorders can have adverse effects on both quality of life and outcomes of cancer treatment. Unrecognized hypothyroidism or hyperthyroidism can be confused with symptoms related to toxicities of therapy and may cause the treating oncologist to reduce a medication dose unnecessarily or put treatment temporarily on hold. Symptoms are often unreliable, and cancer patients should be screened appropriately for underlying thyroid disease.

This article will review the presentation, diagnosis, and management of the most common thyroid disorders as well as special circumstances related to oncologic care. As more patients become long-term cancer survivors, it is important for primary care providers and oncology advanced practitioners to be aware of potential endocrinopathies that may develop during or after cancer treatment. Complex cases should always be referred to a knowledgeable endocrinologist.

## THYROTOXICOSIS/HYPERTHYROIDISM

Hyperthyroidism occurs when there are increased levels of circulating thyroid hormone and a decreased serum TSH level due to Graves’ disease (most common) or toxic goiter. Graves’ disease, which most commonly affects women between the ages of 30 and 50, is an autoimmune condition (Toft & McIver, 2007). Circulating antibodies provoke the thyroid to release too much thyroid hormone. A toxic nodule is an autonomously functioning discrete mass in the gland that does not rely on TSH regulation. Much less commonly, elevated thyroxine levels can be drug-induced (amiodarone, interferon alpha).

**Assessment**

Diagnostic testing includes labs for TSH, free T4, and T3 levels. Thyroid-stimulating immunoglobulins (TSIs) are markedly elevated in Graves’ disease.

Imaging in the form of thyroid uptake and scan will show the pattern of radionucleotide distribution in the gland. Homogeneous uptake is consistent with Graves’ disease, whereas single or multiple discrete foci suggest a toxic nodule/goiter. Toxic nodules are nearly always benign, although case reports have shown that they may harbor thyroid cancer. Recent CT scan with contrast (within 4–6 weeks) could affect the reliability of this study, as the patient may be saturated with iodine already and unable to concentrate any of the isotope in the thyroid.

**Symptoms**

Patients may be asymptomatic with mild hyperthyroidism but can be profoundly symptomatic as T4 and T3 levels rise. Early symptoms may include palpitations or nervousness. Patients may experience increased hunger, heat intolerance, and sweating. There can be increasing frequency of bowel movements (with or without diarrhea). Severe thyrotoxicosis, or thyroid storm, can be accompanied by fever, tachycardia, nausea, vomiting, diarrhea, marked hyperirritability, and anxiety (Braverman & Cooper, 2000).

Clinical signs may include weight loss, tachycardia, new-onset atrial fibrillation, tremor, systolic hypertension, or sweating. Graves’ disease is often accompanied by ophthalmopathy. Swollen, inflamed extraocular muscles may lead to proptosis and the appearance of lid retraction. Patients may complain of blurry or double vision. Ophthalmologic consultation for detailed visual evaluation and protection of the cornea is important. 

**Treatment**

Antithyroid drugs can act as temporizing agents to control circulating thyroid levels by inhibiting the synthesis or release of T4 and T3, but definitive management consists of surgery (thyroidectomy) or radioactive iodine ablation (Braverman & Cooper, 2000).

Methimazole is generally preferred and has a starting dose of 20 to 30 mg orally daily, often in divided doses, although it can be taken just once a day for convenience. Propylthiouracil (PTU) is rarely used anymore due to concerns about hepatotoxicity but is preferred in pregnant and breast-feeding patients due to lower transplacental passage and lower concentration in breast milk compared with methimazole (Braverman & Cooper, 2000). The usual starting dose of PTU is 100 mg orally three times a day.

Beta blockers are used to control the heart rate and reduce the sensation of palpitations. Patients who are severely hyperthyroid (or who need to be rendered euthyroid quickly, such as for surgery) will benefit from saturated solution of potassium iodide (SSKI) and possibly glucocorticoids. Systemic steroids may alleviate acute Graves’ ophthalmopathy, and orbital surgery at a later date may be considered for ongoing symptomatology attributed to exophthalmos.

Side effects of medical therapy include fever, rash, urticaria, increase in liver transaminases, and rarely agranulocytosis (0.2–0.5%; Braverman & Cooper). Complete blood cell count and liver function tests should be measured at baseline prior to starting therapy as well as periodically in patients who are on long-term medical therapy.

Radioiodine therapy is orally administered as a single dose, and the radiation concentrates in thyroid follicular cells and destroys them. Doses given for Graves’ disease are much lower than for treatment of thyroid cancer, and patients are usually discharged home without the need for radioactive precautions.

Pregnancy and breast-feeding are absolute contraindications to radioactive iodine treatment. A relative contraindication is thyroid ophthalmopathy, and caution should be used in these patients (Lee, 2012).

Many Graves’ patients will eventually develop hypothyroidism. Ablation of toxic nodules is not likely to cause hypothyroidism, as the normal surrounding gland will continue to function. Therapeutic radioactive iodine cannot be administered if the patient has had recent iodinated contrast, such as for CT scan or angiography. It often takes 1 to 3 months for the body to eliminate the contrast, depending on renal function. Urine iodine concentration can be measured as a 24-hour collection to confirm that the patient is not iodine-saturated before receiving radioiodine. If radioactive iodine is to be given to a cancer patient, close communication between the oncologist and endocrinologist can prevent inadvertent "contamination" with iodine contrast for staging studies.

Surgery, generally total or subtotal thyroidectomy, is immediately effective and results in lifelong hypothyroidism requiring medication. Risks include general anesthesia, bleeding, and infection. Adverse outcomes specific to the procedure are potential injury to the recurrent laryngeal nerves or parathyroid glands, which could result in hoarseness or chronic hypocalcemia, respectively. Surgery is not the treatment of choice for medically unstable patients or those on systemic chemotherapy whose thyrotoxicosis can be managed by other means.

Thyroid labs should be monitored a couple of weeks after initiation of medical therapy and continued every few weeks until levels stabilize. It is common for the TSH level to remain low or suppressed for many months (especially in patients who have had long-term thyrotoxicosis) as the T4 and T3 levels drop into the normal range. The dose of medication should be decreased as necessary to prevent iatrogenic hypothyroidism.

If patients are controlled on a low dose of antithyroid medication, the drug can be held, and follow-up laboratories will show whether they are in remission from Graves’ disease. (Unlike Graves’ disease, a toxic goiter does not generally go into remission, and definitive management in the form of radioactive iodine or surgery is appropriate for toxic goiter.) In a small study, about half of patients with Graves’ disease remained in remission 1 year after stopping therapy (Braverman & Cooper, 2000), with no evidence of recurrent thyrotoxicosis.

**Subclinical Hyperthyroidism**

Low TSH levels with normal T4 and T3 levels in an asymptomatic patient are indicative of subclinical hyperthyroidism. Diagnostic imaging with a thyroid scan may still be useful to differentiate between Graves’ disease and toxic goiter. These patients can often be followed and may not need intervention. If they progress to overt hyperthyroidism, treatment should be initiated as previously outlined.

## HYPOTHYROIDISM

Low serum thyroid hormone levels are suggestive of hypothyroidism. Patients with hypothyroidism have a normal or low free T4 level and an elevated TSH level (except in the case of pituitary failure). Causes of hypothyroidism are most often primary: destruction of the gland due to autoimmune disease or radiation; surgical removal of the thyroid; or infiltration of the tissue, as in the case of amyloidosis or rarely cancer. There are many medications that are associated with hypothyroidism, and they will be discussed separately (see section on Drug and Radiation Effects).

**Assessment**

Hashimoto thyroiditis is the most common cause of hypothyroidism in North America and is far more common in women than in men, with a ratio of 20:1; it is generally seen in women between the ages of 45 and 65 years (Oyedeji, Giampoli, Ginat, & Dogra, 2013). Hashimoto thyroiditis is a chronic autoimmune phenomenon confirmed by elevated antithyroperoxidase (TPO antibody) levels in serum. Patients with this condition are initially hyperthyroid, although this period is often missed, and they progress to hypothyroidism over time.

Secondary, or central, hypothyroidism is seen in pituitary disease and is caused by a large tumor, surgery, and/or pituitary radiation. In this case, TSH deficiency is causing the low thyroid levels, and therefore both the TSH and free T4 levels are low. Patients with brain tumors or a history of radiation treatment to the pituitary region cannot be adequately assessed with only a TSH level, as a low TSH level due to decreased pituitary production can be misinterpreted as thyrotoxicosis. These patients may also have an inappropriately normal TSH level with frankly low levels of free T4 and would need medical therapy and ongoing monitoring of serum free T4 levels to monitor dosing.

**Symptoms**

Presenting symptoms vary widely and are not always a reliable indicator of the degree of hypothyroidism. Some patients with profound hypothyroidism are relatively asymptomatic, whereas others with mild disease complain of all manifestations: fatigue, cold intolerance, weight gain, constipation, dry skin, arthralgia, and depression. Physical exam findings also vary but can include slow deep tendon reflexes, periorbital or pretibial edema, excessive dry skin, flat affect, and delayed relaxation of reflexes.

**Treatment**

For patients with true hypothyroidism, treatment is generally initiated in the form of T4 replacement with levothyroxine, at a starting dose of 1.6 mg/kg. Subclinical hypothyroidism, in which patients have a mildly elevated TSH level and a normal free T4 level, can be observed in the absence of symptoms or comorbidities. Exceptions may be made for patients with a history of neck radiation or who are taking high-risk medications such as tyrosine kinase inhibitors, interferon, or lithium. These patients are more likely to progress to overt hypothyroidism if left untreated.

Goals of therapy with levothyroxine are to normalize laboratory values and improve symptoms. Due to the long half-life of this drug, it is recommended that follow-up labs are checked no sooner than 4 to 6 weeks after the initiation of therapy. Patients with extremely elevated TSH levels (greater than 50 mIU/L) may take longer to normalize. If their serum free T4 level is on the higher end of the reference range after 4 to 6 weeks of medical therapy, their TSH levels may continue to fall over subsequent weeks, and it would be reasonable to delay any dose adjustment until another set of lab studies is obtained.

Patients should be counseled to take their thyroid pill first thing in the morning on an empty stomach and not with other medications. For patients who are dependent on a feeding tube, levothyroxine can be crushed and administered with water via the tube. In these cases, a higher dose of medication is usually required to achieve a normal TSH level compared with what would be sufficient if taken orally.

Nonthyroidal illness, sometimes known as sick euthyroid syndrome, is a frequent reason for inpatient endocrine consultation. Major illness induces changes in all anterior pituitary hormone secretion, and patients undergoing treatment for cancer often undergo surgery or chemotherapy, which would make them particularly likely to be affected.

Laboratory tests drawn during acute or chronic illness may show low serum T3 levels; T4 levels are generally normal in these patients, except in the most critically ill patients. Levels of TSH are normal to low but may become elevated during recovery (Braverman & Cooper, 2000). If thyroid laboratory tests are drawn during a period of serious illness and are abnormal, it is sometimes difficult to confirm whether there is a true hormonal disorder. Repeating the blood tests when the patient has recovered will often reveal these levels have returned to normal.

## DRUG AND RADIATION EFFECTS

In this era of targeted therapies, many patients are on oral systemic therapy for cancer treatment. Additionally, some drugs used in the primary care setting have the potential to cause hormonal abnormalities. Radiation treatment is utilized for a variety of malignancies and can lead to delayed consequences with regard to thyroid function.

Targeted tyrosine kinase inhibitors (TKIs) are used in the treatment of renal cell cancers, pancreatic neuroendocrine tumors, hepatocellular carcinoma, and gastrointestinal stromal tumors (GIST). They are also in clinical trials as potential therapeutic options for many other solid tumors. These drugs target growth factor receptors and shorten cell survival (Ahmadieh & Salti, 2013).

The TKIs can induce thyroid dysfunction, especially notable with sunitinib (Sutent), which has a reported association of 32% to 85% (Ahmadieh & Salti, 2013; Hamnvik, Larsen, & Marqusee, 2011). In a prospective study, the median time to develop thyroid dysfunction in patients receiving sunitinib was 4 weeks, but the onset is variable (Ahmadieh & Salti, 2013; Hamnvik, Larsen, & Marqusee, 2011). Possible mechanisms include induction of thyroiditis, capillary regression in the thyroid gland, antithyroid peroxidase antibody production, and decreased iodine uptake by the thyroid gland. Some patients had transiently suppressed TSH levels, suggestive of thyroiditis as the etiology of their hypothyroidism.

Thyroid function should be monitored regularly in patients who are on TKI therapy, and appropriate treatment should be initiated if indicated. Other TKIs associated with a variable risk of new-onset hypothyroidism include sorafenib (Nexavar), imatinib, dasatinib (Sprycel), nilotinib (Tasigna), and axitinib (Inlyta; Hamnvik, Larsen, & Marqusee, 2011). Drug-induced hypothyroidism can persist after the conclusion of TKI therapy. Additionally, patients already on thyroid hormone often have increased dose needs after starting TKI therapy. Interestingly, there appears to be a relationship between development of hypothyroidism in patients with metastatic renal cell carcinoma and improved outcomes (Bianchi et al., 2013).

Cytokines, including interferons and interleukins, are also known to affect thyroid hormone secretion and metabolism (Braverman & Cooper, 2000). Interferon-alpha is used for treatment of hepatitis C, melanoma, renal cell cancer, and some hematologic malignancies and has been associated with destructive or autoimmune thyroiditis, leading to persistent hypothyroidism after a brief thyrotoxic phase. The risk of hypothyroidism has been reported to be 2% to 10%, with a median onset of 4 months (Hamnvik, Larsen, & Marqusee, 2011).

A multitude of non-oncologic drugs are associated with a risk of hypothyroidism. Lithium is occasionally used for treatment of bipolar disorder and other psychiatric conditions. It inhibits thyroid hormone secretion and can lead to subclinical (34%) or overt (15%) hypothyroidism (Braverman & Cooper, 2000). Annual laboratory screening with TSH and free T4 levels is appropriate. Amiodarone is an iodinated drug used for arrhythmia that can lead to hyperthyroidism or hypothyroidism due to a number of effects on thyroid hormone uptake and deiodination. Rifampicin induces hepatic metabolism of T4 and increases its rate of clearance (Braverman & Cooper, 2000). Patients on treatment for tuberculosis, and especially those already taking supplemental levothyroxine, require monitoring during therapy.

Drug-induced central hypothyroidism is much less common than drug-related primary hypothyroidism. Ipilimumab (Yervoy) is a monoclonal antibody approved for treatment of unresectable or metastatic melanoma that may lead to increased T-cell activation and antitumor effects. Ipilimumab causes lymphocytic hypophysitis (autoimmune destruction of the pituitary gland). These patients have impaired anterior pituitary function, which usually includes low levels of gonadotropins and adrenocorticotropic hormone (ACTH) deficiency as well as central hypothyroidism.

The incidence of hypophysitis is not well defined, but it may be up to 17% (Hamnvik, Larsen, & Marqusee, 2011). Bexarotene (Targretin) is currently approved for treatment of cutaneous T-cell lymphoma and is also under investigation for other cancers, such as lung, breast, and thyroid. It can cause isolated TSH deficiency with no other hormonal abnormalities.

**Radiation**

Ionizing radiation to the neck is a known risk factor for the development of thyroid nodules, thyroid cancer, and primary hypothyroidism, particularly when exposure is during childhood. Hypothyroidism is one of the late side effects seen after curative radiotherapy in the head and neck region. Five years after curative radiotherapy, 48% of patients develop hypothyroidism, with a median onset at about 1.5 years posttreatment (Boomsma, Bijl, & Langendijk, 2011). Among other factors, the risk of hypothyroidism is related to both the total dose given as well as the percentage of thyroid gland in the field.

Radiation exposure to the pituitary can cause damage to the gland and a gradual drop in TSH production leading to central hypothyroidism. This type of damage would generally cause other anterior pituitary hormone deficiencies as well.

In a study of nasopharyngeal cancer patients, exposure of both thyroid and pituitary glands to radiation was assessed, with a cumulative dose of more than 50 Gy considered to be "high." After 18 months of follow-up, patients who received a high dose of radiation to both glands had an 83% incidence of hypothyroidism (central or primary). Those in the high-dose thyroid group with low pituitary exposure had a 50% risk. Of the 65 patients studied, 23% had overt or subclinical hypothyroidism at the 18-month assessment (Lin et al., 2013).

## GOITER/NODULES

Thyroid nodules are common and are detected on clinical examination in 4% to 8% of adults, by ultrasonography in up to 70%, and in 50% of autopsies (Oyedeji et al., 2013; Hambleton & Kandil, 2013). Incidental thyroid nodules have an estimated risk of malignancy of approximately 5% (Boeckmann, Bartel, Siegel, Bodenner, & Stack, 2012). A goiter, or diffusely enlarged thyroid gland, is often palpable but usually asymptomatic. Large goiters may cause changes in voice, difficulty swallowing, or rarely difficulty breathing.

^18^F-fluorodeoxyglucose–positron emission tomography (FDG-PET) is a valuable imaging modality in oncology. Tumor cells have higher metabolic activity and concentrate ^18^FDG more than normal tissue, which is useful in providing information for diagnosis (Figure), tumor staging, surveillance, and monitoring response to therapy (Boeckmann et al., 2012).

**Figure 1:**
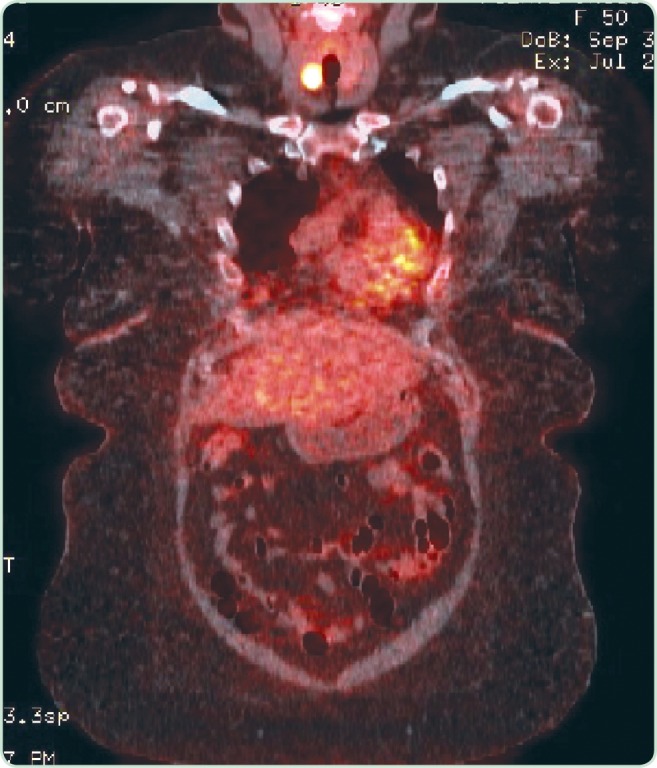
Incidental PET-avid right thyroid nodule detected on staging for colon cancer. Pathology assessment later confirmed primary thyroid cancer.

In one review of more than 20,000 PET scans performed for characterization of various malignancies, the incidence of ^18^FDG uptake in the thyroid gland was about 5%. Half of them were focal, and the other half were diffuse in appearance. Diffuse uptake is generally benign and due to thyroiditis. Focal uptake has a reported risk of malignancy of anywhere from 23% to 50% (Boeckmann et al., 2012) and requires further evaluation with ultrasonography and possible biopsy.

Of those that are malignant, most are of thyroid origin (most commonly papillary thyroid cancer), with the remainder being thyroid metastases from other primary tumor sites. Higher maximum standardized uptake values (SUV) have been associated with a greater risk of malignancy (Boeckmann et al., 2012) but are not sufficient for determining the nature of the thyroid abnormality.

**Assessment**

Thyroid nodules are best evaluated with neck ultrasonography. Most of these nodules are benign, but several characteristics should prompt fine-needle aspiration (FNA) to confirm there is no malignancy present. Suspicious characteristics include marked hypoechogenicity compared with the surrounding thyroid parenchyma, ill-defined borders, hypervascularity, and microcalcifications (Oyedeji et al., 2013). Abnormal-appearing lymph nodes adjacent to the thyroid gland should also be assessed with FNA to confirm that there is no occult thyroid malignancy or metastasis present from another anatomic site.

## NONTHYROID MALIGNANCIES IN THE THYROID GLAND

Primary thyroid lymphoma is an uncommon presentation of lymphoma (< 5%) and, when it occurs, is often the non-Hodgkin variant (Oyedeji et al., 2013). Most patients who develop these malignancies are older than age 50. Diffuse lymphadenopathy on physical examination or imaging studies suggests this diagnosis. The thyroid will appear enlarged and heterogeneous on ultrasonography, often with internal vascularity. PET scan will show avid uptake of radiolabeled glucose. Thyroid metastases are rare but should be considered in patients with advanced or diffuse malignancy and a new thyroid mass. The most common malignancies to metastasize to the thyroid are breast, lung, and renal cell carcinomas, with renal tumors being the most common (Oyedeji et al., 2013).

**Treatment**

Benign thyroid nodules (detected either by ultrasound criteria or FNA) can be observed with follow-up ultrasonography in 1 year. A small percentage of thyroid nodules will be undiagnosed after ultrasonography and sometimes FNA. They include suspicious-appearing lesions that are not accessible to biopsy due to location as well as indeterminate/inconclusive findings on needle biopsy (insufficient sampling, follicular lesion, or follicular neoplasm).

Management of these patients should be considered in the context of their cancer status. For patients with advanced malignancies or who are undergoing cytotoxic chemotherapy (or who have other significant comorbidities), short-term observation with follow-up ultrasonography can be performed. Patients who are otherwise healthy and have a good performance status should be considered for surgical excision to better characterize the nodule, although sonographic follow-up is also appropriate. In the case of insufficient sampling, repeat FNA at the time of follow-up ultrasonography in 6 to 12 months can be attempted. Diagnostic molecular studies are becoming more widely available and can be helpful in estimating the risk of malignancy when FNA cytology is "follicular" in nature.

Surgery for benign-appearing thyroid masses should be limited to patients with symptoms, such as dyspnea, dysphagia, or hoarseness. If FNA is benign, but there is clinical concern (i.e., enlarging nodule or other suspicious findings), hemithyroidectomy can be performed for the purpose of establishing a diagnosis. This technique decreases the risk of complications, such as recurrent laryngeal nerve injury or hypoparathyroidism. Many patients maintain a euthyroid state after hemithyroidectomy and do not require supplemental thyroid hormone.

## CONCLUSIONS

Advanced practitioners (APs) are likely to see patients with a history of cancer and a coexisting thyroid problem. Cancer treatments, such as radiation and certain medications, can cause hypothyroidism. In other cases, a new issue is incidentally discovered during workup or staging of their malignancy. Proper evaluation and management are key to maintaining the patient’s quality of life as well as optimizing the outcome of their cancer treatment. Many chronic thyroid issues can be followed by the patient’s primary provider in coordination with their oncologist or endocrinologist. It is important for all APs to be aware of the presentation and symptoms associated with thyroid hormone abnormalities so that these conditions can be recognized and addressed.
